# Peptide–polymer ligands for a tandem WW-domain, an adaptive multivalent protein–protein interaction: lessons on the thermodynamic fitness of flexible ligands

**DOI:** 10.3762/bjoc.11.93

**Published:** 2015-05-18

**Authors:** Katharina Koschek, Vedat Durmaz, Oxana Krylova, Marek Wieczorek, Shilpi Gupta, Martin Richter, Alexander Bujotzek, Christina Fischer, Rainer Haag, Christian Freund, Marcus Weber, Jörg Rademann

**Affiliations:** 1 Institute of Pharmacy & Institute of Chemistry and Biochemistry, Freie Universität Berlin, Königin-Luise-Str. 2+4, 14195 Berlin, Germany; 2 Department of Medicinal Chemistry, Leibniz Institut für Molekulare Pharmakologie, Robert-Rössle-Str. 10, 13125 Berlin, Germany; 3 Fraunhofer Institute for Manufacturing Technology and Advanced Materials (IFAM), Wiener Str. 12, 28359 Bremen, Germany; 4 Konrad-Zuse-Zentrum für Informationstechnik Berlin, Numerical Analysis and Modelling, Takustr. 7, 14195 Berlin, Germany

**Keywords:** inhibitors of protein–protein interactions, isothermal titration calorimetry, multivalency, peptide–polymer conjugates, proline-rich peptide sequences

## Abstract

Three polymers, poly(*N*-(2-hydroxypropyl)methacrylamide) (pHPMA), hyperbranched polyglycerol (hPG), and dextran were investigated as carriers for multivalent ligands targeting the adaptive tandem WW-domain of formin-binding protein (FBP21). Polymer carriers were conjugated with 3–9 copies of the proline-rich decapeptide GPPPRGPPPR-NH_2_ (**P1**). Binding of the obtained peptide–polymer conjugates to the tandem WW-domain was investigated employing isothermal titration calorimetry (ITC) to determine the binding affinity, the enthalpic and entropic contributions to free binding energy, and the stoichiometry of binding for all peptide–polymer conjugates. Binding affinities of all multivalent ligands were in the µM range, strongly amplified compared to the monovalent ligand **P1** with a *K_D_* > 1 mM. In addition, concise differences were observed, pHPMA and hPG carriers showed moderate affinity and bound 2.3–2.8 peptides per protein binding site resulting in the formation of aggregates. Dextran-based conjugates displayed affinities down to 1.2 µM, forming complexes with low stoichiometry, and no precipitation. Experimental results were compared with parameters obtained from molecular dynamics simulations in order to understand the observed differences between the three carrier materials. In summary, the more rigid and condensed peptide–polymer conjugates based on the dextran scaffold seem to be superior to induce multivalent binding and to increase affinity, while the more flexible and dendritic polymers, pHPMA and hPG are suitable to induce crosslinking upon binding.

## Introduction

**Figure 1:**
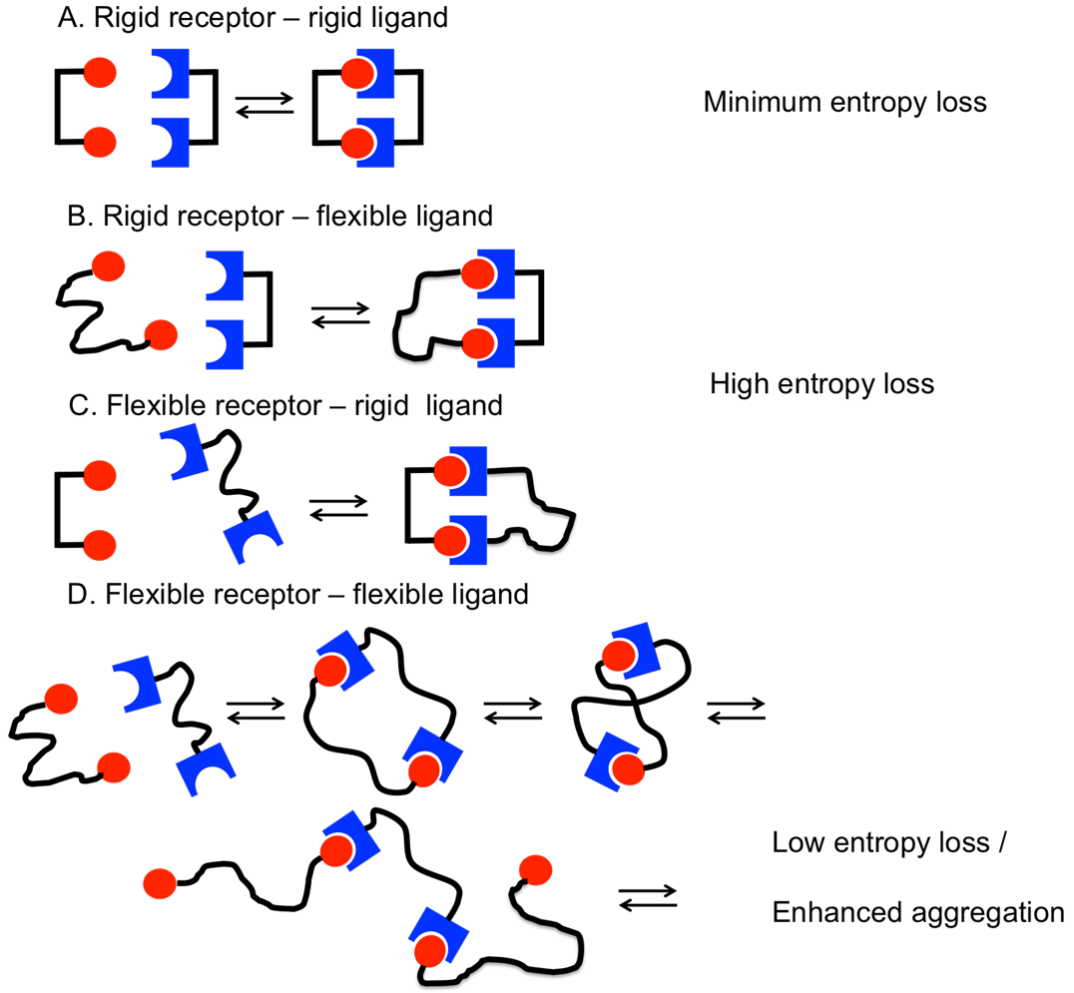
Comparing the entropy loss during ligand–receptor interactions in dependence of the rigidity of the backbone.

Multivalency is a general principle in nature for increasing the affinity and specificity of ligand–receptor interactions [1]. Multivalent binding is characterized by the cooperative, over-additive enhancement of binding affinities of ligands and receptors in a defined spatial arrangement. The strongest affinity enhancement can be expected in the case of a perfectly fitting, rigid arrangement of ligands and receptors (Figure 1A). In such cases the affinity of the multivalent ligand can be potentiated by the degree of multivalency. Prominent examples for this perfect fit have been reported reaching an exponential binding increase [2]. Rigid scaffolds can be used to present ligands in defined spatial arrangements and thus can be exploited to investigate the distances between receptor sites as “molecular ruler” [3–4].

Many multivalent receptors in nature, however, are characterized by the flexible arrangement of receptor sites and the resulting relative mobility of binding domains seems to have a significant impact on the proper functioning of these proteins [5]. Flexible arrangements of receptor sites can result from different scenarios. In many proteins flexibility is introduced by regions of inherent structural mobility, e.g., by so-called unstructured regions inserted between the receptor domains of a multireceptor protein. Alternatively, the relative mobility of binding sites is realized by their embedding into membranes giving them a certain degree of freedom to move in the plane of the membrane, or by incorporation into dynamic multiprotein complexes.

Design of potent multivalent ligands for flexible receptor arrangements is a considerable challenge, as the flexibility of multivalent ligands and the flexibility of receptors have to be matched in order to balance enthalpic gain with entropic loss of the system. In such a setting, a rigid multivalent ligand binding to a flexible receptor can be expected to reduce the entropy of the system upon binding, and thus will result in a partial or complete loss of the multivalent affinity enhancement. For example, the targeting of flexible protein receptors with ligands attached to a rigid DNA-backbone has been reported to be unsuccessful and no preferred ligand distance was found for this “molecular ruler” for flexible divalent protein targets [4].

Recently, we have introduced multivalent peptide–polymer conjugates as a chemical tool to inhibit protein–protein interactions in living cells [6]. As demonstrated for the pro-apoptotic BH3-peptides, multivalent presentation of monovalent ligand peptides can potentiate the activity of the peptide at identical overall peptide concentrations. Moreover, attachment of bioactive peptides to polymers strongly enhanced their stability and protected them from proteolysis [7–8]. The construction of peptide-polymer conjugates with additional cell-penetrating peptides attached [9] enabled the smooth intracellular delivery of the conjugated polymer; as a third component fluorescent dyes [10] were coupled to the polymers simultaneously with the bioactive and the cell-penetrating peptides in order to enable the monitoring of cellular uptake and intracellular distribution of the peptide–polymer conjugate.

Until now, various polymer carriers have been used for the construction of peptide–polymer conjugates [11–12], however, a systematic comparison of the different polymeric materials with respect to the structure–activity relationships is missing so far. The goal of this contribution is to synthesize and compare flexible multivalent ligands for an adaptive, divalent receptor as a protein target. As a model protein the tandem-WW-domain of the pre-mRNA splicing factor formin binding protein 21 (FBP21) was selected [13–15]. Considering the importance of FBP21 in the activation of RNA splicing, successful ligands should be valuable tools to interfere with FBP21-dependent splicing events. Several multivalent ligands were synthesized on the basis of various polymer supports differing in their chemical structure, backbone flexibility, morphology, and ligand loading. The obtained materials were then investigated in order to contribute to the understanding of structure–activity relationships of polymeric ligands. For this purpose, the thermodynamics and the stoichiometry of protein binding events were determined experimentally for all multivalent ligands. Finally, atomistic molecular dynamics simulations were conducted in order to rationalize the observed differences on a microscopic level and to derive general principles for the design of optimized multivalent ligands of flexible protein targets.

## Results and Discussion

### Selection of a bivalent protein receptor as a target

As a representative example for a protein containing a bivalent domain architecture connected with a flexible linker the tandem WW-domains of the protein FBP21 were selected. FBP21 is a protein component of the spliceosome, the multiprotein complex in the nucleus of cells responsible for the processing of primary RNA-transcripts. The two WW domains of FBP21 bind to proline-rich sequences contained in numerous proteins including the core splicing protein SmB/B´and several splicing factors including splicing factor 3B4 (SF3B4) [16–17]. Recently, the enhanced binding affinity of bivalent and tetravalent peptide ligands to this protein was described suggesting that multivalent ligands may play a significant role also in living cells. In addition, several interaction partners of FBP21 have been profiled by SILAC/MS [18]. As monovalent peptide ligands for each of the two WW domains proline-rich sequences (PRS) of the group R_b_ have been identified, in which the proline residues are flanked by arginine (R in one-letter-code) [16,19]. Multivalent arrangements of these monovalent ligands therefore could serve as potent inhibitors of FBP21-interactions and could be used for the inhibition of FBP21 function. As a monovalent peptide ligand the decapeptide amide GPPPRGPPPR-NH_2_ (**P1**) was selected and synthesized on Rink amide polystyrene resin. For attachment to the polymer carriers the N-cysteinylated peptide CGPPPRGPPPR-NH_2_ (**P2**) was prepared, containing a free N-terminus in order to enable the attachment to polymers via native chemical ligation or Michael addition to maleimide residues.

### Selection of polymer carriers and synthesis of multivalent ligands

**Scheme 1:**
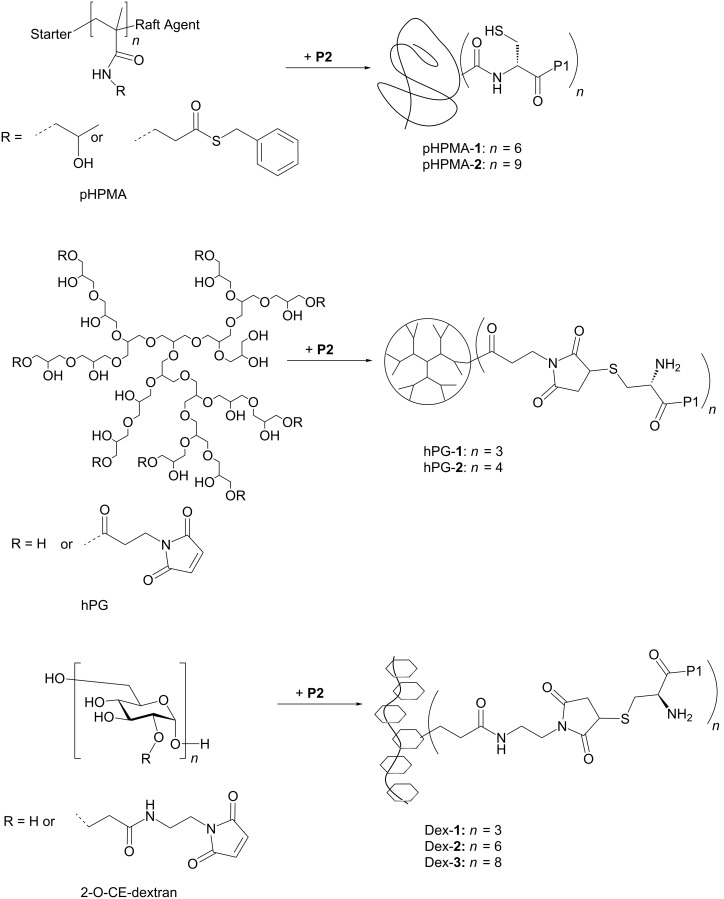
Selection of three polymer carriers differing with respect to backbone flexibility, and morphology and used for the construction of peptide–polymer conjugates.

Three biocompatible polymers with different chemical structure, backbone flexibility and polymer morphology were selected as multivalent ligand carriers, two linear polymers and one dendritic polymer (Scheme 1). Linear poly(*N*-(2-hydroxypropyl)methacrylamide) (pHPMA) possesses a C2 repeating unit with three fully rotatable bonds, which should convey – compared to the other polymers employed in this study – high backbone flexibility to this carrier. Reactive pHPMA was prepared in a copolymerization of HPMA and the thioester-containing building block *N*-methacryloyl-β-alaninyl-*S*-benzyl thioester under reversible addition–fragmentation chain-transfer (RAFT) conditions yielding a thioester-containing copolymer with 13.3 kDa and polydispersity of 1.2, which we denominated as NCL-polymer [10]. NCL-polymer was converted into multivalent peptide–polymer conjugates pHPMA-**1** and pHPMA-**2** via native chemical ligation with the N-cysteinylated peptide CGPPPRGPPPR-NH_2_ (**P2**). In contrast, the second carrier molecule, hyperbranched polyglycerol (hPG) was selected as a dendritic polymer. While the backbone of PG is relatively flexible by itself, the dendritic structure of hPG can be expected to limit the flexibility of attached ligands compared to a linear polymer and might induce a more globular arrangement of the ligands. The hPG polymer carrier was synthesized via an anionic ring-opening polymerization of glycidol [20] and also modified with maleimido groups by reaction with *N*-3-chloropropyl maleimide for ligand attachment.

Finally, dextran, a polysaccharide containing α-1,6-linked D-glucose as repeating unit, was selected as the second linear carrier. The D-glucose units in the polysaccharide are fixed in the _1_C^4^ chair conformation and thus can be expected to rigidify the polymer backbone compared to the other two polymers, leaving only two freely rotatable bonds per building block. Structural studies with dextran suggested a helical structure as the lowest energy conformations of this polymer [21]. Dextran was used as a linear polymer with an average *M*_W_ of either 10 kDa (for Dex-**1** and Dex-**2**) or 50 kDa (for Dex-**3**), both with a polydispersity index of 1.5. Under basic conditions the linear polysaccharide was alkylated with acrylamide selectively in the 2-positions of the sugars. The resulting 2-O-carboxyethyl dextran (2-O-CE-dextran) was further converted by condensation with 2-*N*-maleimido-ethylamine and *N*-ethyl-*N*´-dimethylaminopropylcarbodiimide (EDC) [6]. The monovalent ligand peptide **2** was attached to the dextran carriers by nucleophilic addition of the thiols to the maleimide double bond furnishing peptide–polymer conjugates Dex-**1**, Dex-**2**, and Dex-**3**.

Peptide loadings of all obtained peptide–polymer conjugates were determined by quantitative amino acid analysis and ranged from 3 to 9 peptides per polymer corresponding to peptide loading densities (percentage of ligand-carrying monomers) between 3 and 10%.

### Binding of multivalent peptide–polymer conjugate to the tandem WW domain

Binding studies with peptide–polymer conjugates were conducted employing isothermal titration calorimetry (ITC). This method enables the determination of the binding affinity of the multivalent ligands and elucidates the composition of the free energy of binding from the enthalpic and entropic contributions. In addition, the method can be used to determine the stoichiometry of the formed protein–ligand complex indicating the ratio of peptide ligand molecules relative to each protein binding site thereby giving valuable insights into the multivalency of binding and/or the degree of crosslinking. Thus, the method enables the identification of polymer–protein aggregates containing several polymers and proteins in a complex. No precipitation of the multicomponent aggregates that interfered with ITC measurements was observed during the experiments.

**Figure 2:**
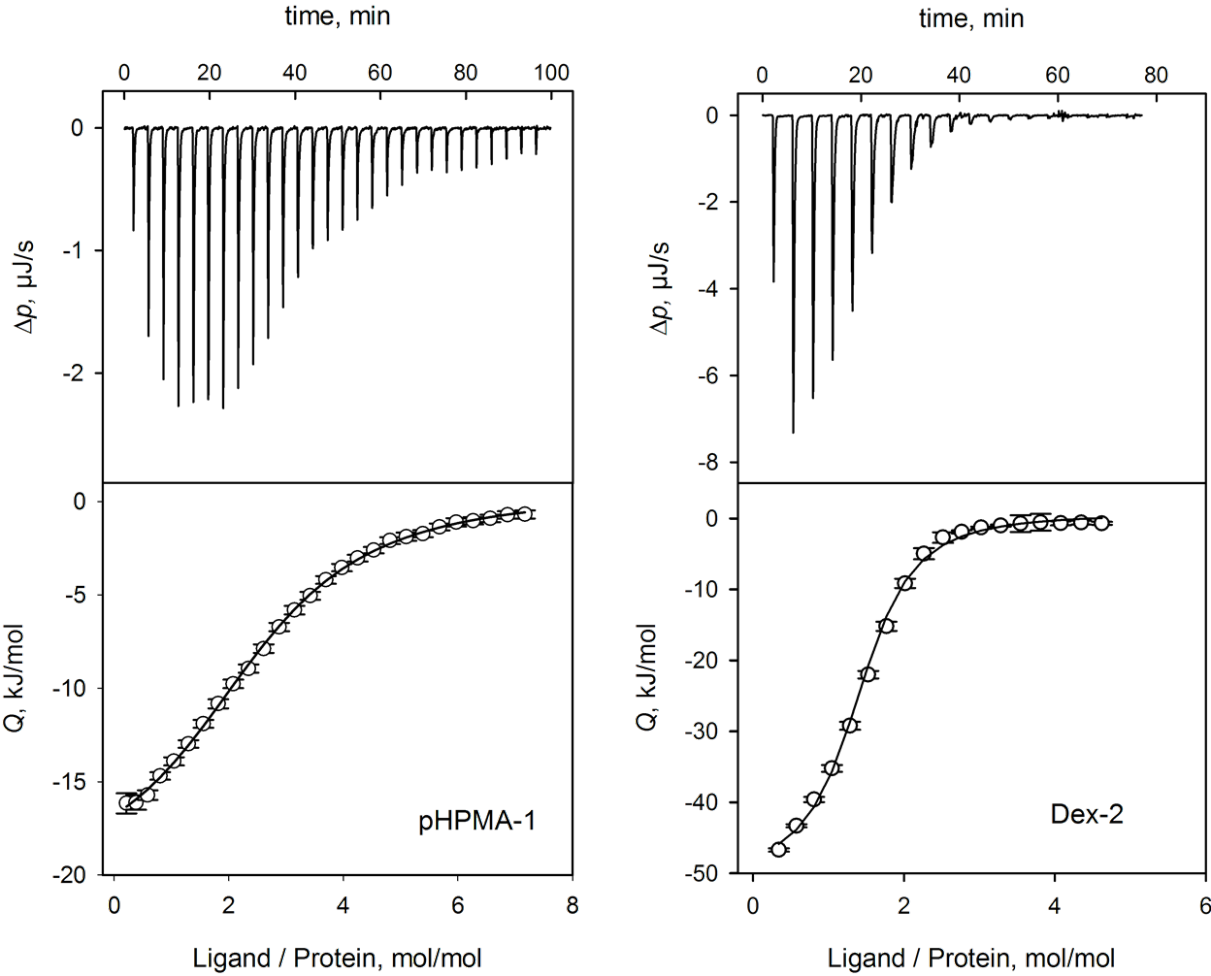
Representative ITC-measurements conducted at 8 °C with the peptide–polymer conjugates A) pHPMA-**1** and B) Dex-**2** showing an increase in affinity for the interaction of Dex-**2** with the FBP21 tandem WW domains. The upper part shows differential heating power (Δ*p*) changes upon injection of peptide–polymer conjugates into the protein; bottom part shows integrated and normalized heat of reaction plotted against peptide/protein molar ratio; binding isotherms are fitted with a 1:1 binding model.

**Figure 3:**
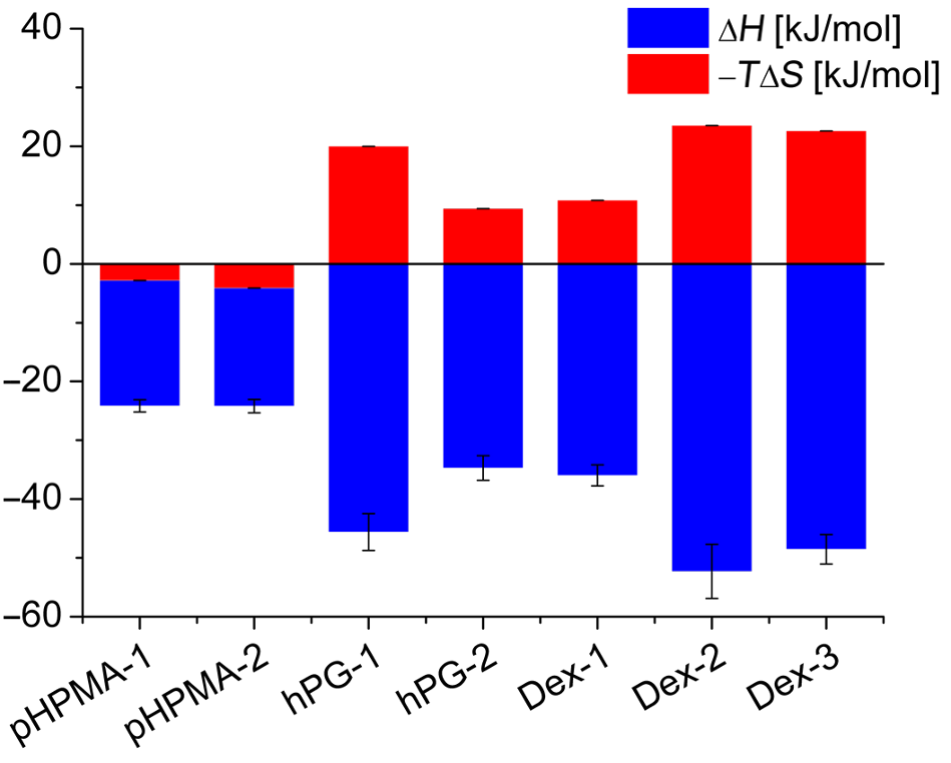
Enthalpic and entropic contributions to the free energy of binding processes of multivalent peptide-polymer conjugates and the tandem WW domain of protein FBP21 determined at 8 °C by ITC measurements.

ITC-analysis (Figure 2) of the binding of multivalent peptide–polymer conjugates yielded *K*_D_ values either corresponding to the polymer concentration or relative to the overall peptide concentration (N*K_D_). A comparison of the binding affinity of the monovalent peptide ligand **P1** and its N-acetylated derivative Ac-**P1** with seven multivalent peptide ligands to the tandem WW-domain revealed a strong enhancement of the binding through multivalency (Table 1, Figure 3). While the peptide alone bound with a dissociation constant (*K*_D_) of larger than 1 mM [16], all multivalent peptide-polymer conjugates possessed *K*_D_ values below 10 µM. Though all *K*_D_ values of multivalent ligands were in the same concentration range (i.e., between 1.2 and 7 µM), concise differences were revealed for the thermodynamic composition of *K*_D_ values (Figure 2). While the ligands based on polymethacrylamide displayed moderate enthalpic and almost negligible entropic contributions , all polyhydroxy-based peptide–polymer conjugates showed significantly stronger generation of heat through binding (enthalpy) together with a pronounced loss in entropy. Binding in all cases was driven mainly by enthalpy, which clearly outweighed the observed entropy loss. In the seven peptide–polymer conjugates investigated, increased loading density of ligands led consistently to increased affinity of the multivalent ligand (Table 1). The most significant difference between dextran and the two other polymer carriers was the stoichiometry of the formed peptide-polymer–protein complex. Inspection of the test solution revealed the formation of a colloidal suspension/dispersion both for pHPMA and for hPG-based peptide conjugates indicating the formation of insoluble aggregates possibly generated through crosslinking. Corresponding to the observed colloidal suspension/dispersion the stoichiometry of peptide ligands per protein receptor resulting from the ITC experiments was >2 for each of either pHPMA or hPG-based material, most pronounced for pHPMA with *n* = 2.6–2.8. Dextran-based conjugates displayed a ligand stoichiometry of 1.4 for the most potent multivalent ligand with a *K*_D_ of 1.2 µM, Dex-**2**. No correlation between ligand density and stoichiometry became evident from the recorded data, however, the observed correlation between low binding stoichiometry, increased binding affinity, and increased binding enthalpy seems to suggest the prevalence of a bivalent binding mode for the complex of Dex-**2** and tandem-WW-FBP21, which is supported also by the solubility of the non-crosslinked peptide-polymer–protein complex.

**Table 1:** ITC measurements of peptide–polymer conjugates with tandem WW domain of FBP21.

Conjugates^a^	N Ligands (rep. units)^b^	Loading density [%]	*K*_D_ [μM]^c^	Rel. *K*_D_ = N*K_D_ [µM]^d^	Binding stoichiometry	Aggregates

**P1**	–	100	> 1000	> 1000	–	–
Ac-**P1**	–	100	>1000	>1000	–	–
pHPMA-**1**	6 (92)	6.5	5.0 ± 0.8	30 ± 5	2.6	X
pHPMA-**2**	9 (108)	8	3.3 ± 0.6	30 ± 5	2.8	X
hPG-**1**	3 (97)	3	6.3 ± 1.7	19 ± 5	2.3	X
hPG-**2**	4 (97)	4	5.0 ± 1.3	20 ± 5	2.4	X
Dex-**1**	3 (62)	5	7.0 ± 1.2	21 ± 4	1.8	–
Dex-**2**	6 (62)	10	1.2 ± 0.7	7 ± 4	1.4	–
Dex-**3**	8 (248)	3	1.6 ± 0.4	13 ± 3	1.3	–

^a^Dextran, hyperbranched PG and poly(HPMA) coupled with the N-cysteinylated peptide CGPPPRGPPPR (**P2**); ^b^*N*: number of ligands (number of repeating units in the polymeric scaffolds); ^c^binding affinities of peptide–polymer conjugates; ^d^binding affinities measured by ITC related to overall peptide concentrations.

### Molecular dynamics simulations of multivalent ligands

**Figure 4:**
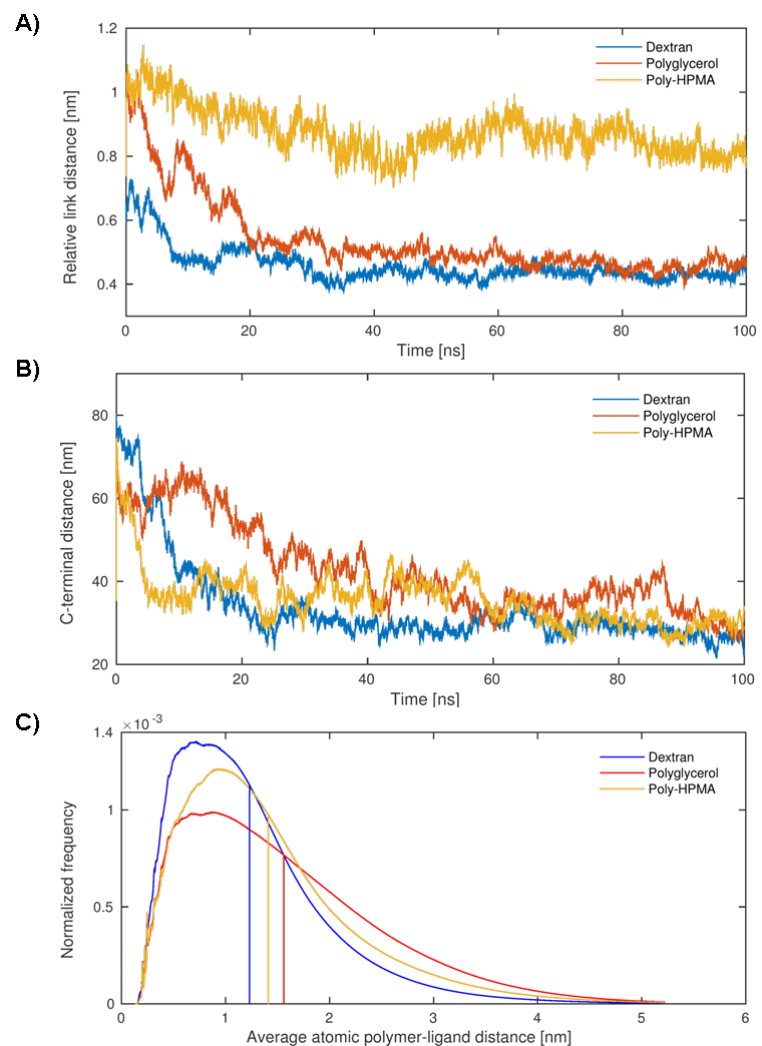
MD simulations over time (0–100 ns) yielding A) the mean sulfur distance between two peptides at their linking site, B) the mean nitrogen distance between two peptides at the farthest distance between peptide and polymer chain C) the frequency of observed peptide–polymer distances in dependence of the polymer backbone pHPMA, hPG and dextran, respectively.

In order to better understand our experimental observations regarding binding affinities, enthalpic/entropic energy contributions, and binding stoichiometries from a molecular point of view, the three polymer carriers were investigated using atomistic molecular dynamics simulations. Each polymer was represented by one model parameterized in accordance with the AMBER force field [22]. The concentration ratios of peptide ligands and monomeric units were fit to lab conditions such that each polymer was carrying three ligands. In contrast to the linear polymer models of dextran and pHPMA with 10 and 12 units between any two successive ligands, respectively, the hPG configuration was generated randomly with the aid of a probabilistic hPG building algorithm as described previously [23]. After some preparatory relaxation steps, each of the three polymers underwent three explicit solvent molecular dynamics (MD) simulations of 100 ns length serving as production runs. The first 30% of the time steps were considered as an unrestricted equilibration phase and consequently omitted whereas from the remaining time series several promising structural and physical descriptors were determined. For all simulations and analytical calculations the Gromacs software suite was utilized [24]. Table 2 and Figure 4 show these theoretical results averaged over time as well as the three runs per polymer.

**Table 2:** Molecular dynamics simulations of the protein target and the multivalent polymeric ligands.

Polymeric scaffold	pHPMA	hPG	Dextran

Mean distance (expected value) rdf [nm]^a^	1.41	1.56	1.23
Peptide distance at binding site [nm]^b^	0,84	0,48	0,43
Peptide distance at the termination site [nm]^c^	3,39	3,66	2,9
*E*(peptide-polymer) [kJ/mol]^d^	−515,3	−783,3	−912,7
*E*(peptide-solution) [kJ/mol]^e^	−3268,7	−3224,8	−3281,1
Globularity^f^	0,037	0,104	0,066

^a^Expected mean distance values (calculated by a radial distribution function); mean distance between two peptide ligands on a polymer chain measured between ^b^the N-terminal sulfur atoms of the Cys-residues at their linking site and ^c^the C-terminal nitrogen atoms of the Arg residue as the farthest distance between peptide and polymer backbone; average potential energy regarding ^d^the affinity of the peptide to the polymer and ^e^the solvation energy of the peptide; ^f^ratio of the peptide-polymer conjugates volume and the appropriate sphere.

**Figure 5:**
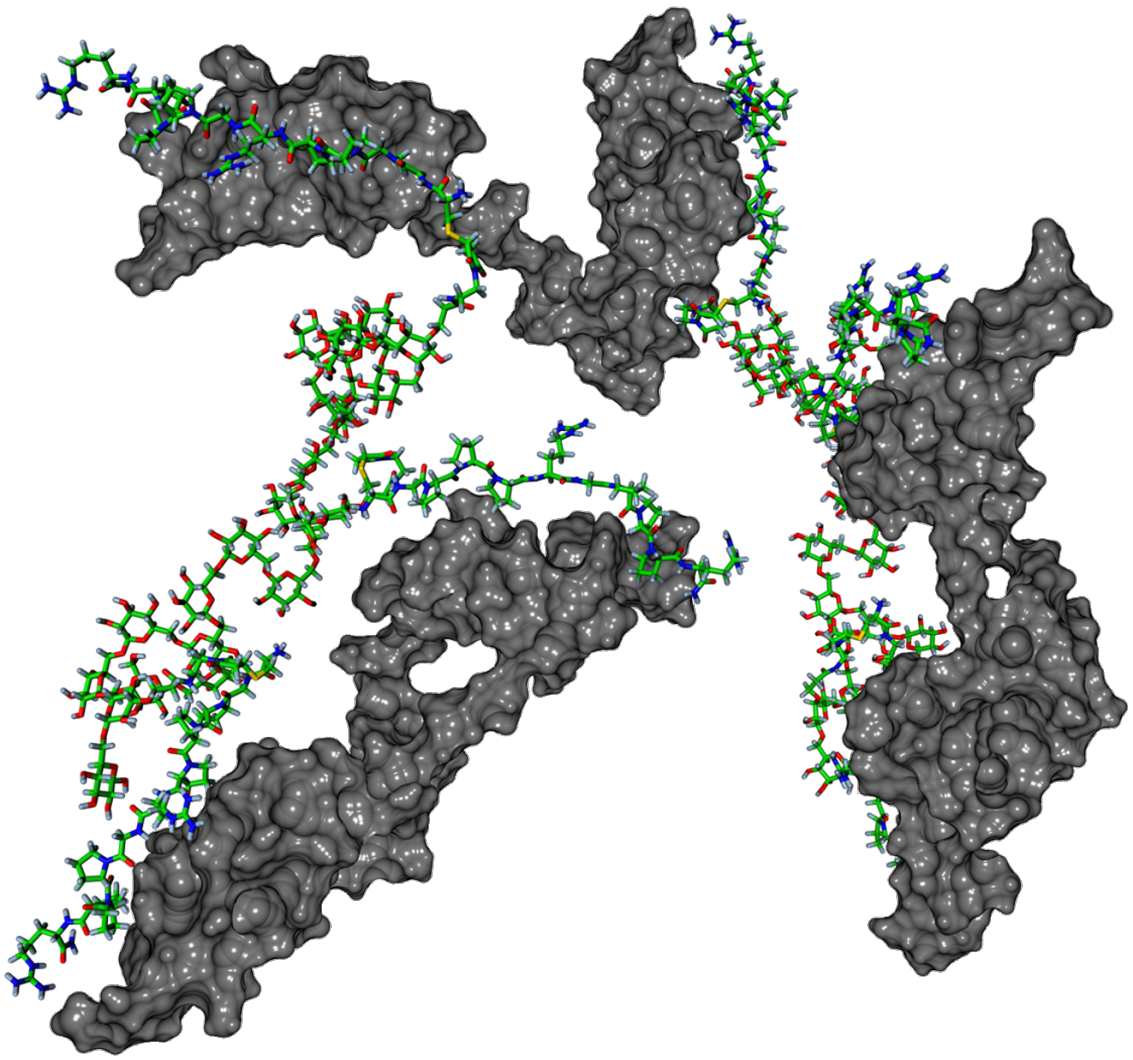
MD simulation image showing the interaction of two dextran–peptide conjugates with three tandem WW domains of FBP21 illustrating the intramolecular mode of binding.

**Structural properties and descriptors.** Dividing the Euklidean distance between two successive peptide attachment points by the number of bonds in between (i.e., between the N-terminal nitrogen atoms of the cysteinylated peptide **P2** in the case of pHPMA, and the Cys-sulfur in the cases of both hPG and dextran) yields relative distances which indicate that the peptide ligands in pHPMA are further apart than in dextran and hPG, while the variance of the peptide positions in pHPMA is higher than in the two hydroxyl polymers (Table 2, Figure 4A). Next, we were interested in the distances between the C-terminal positions of the peptide ligands measured between the C-terminal amide nitrogens of the peptides (Table 2, Figure 4B). Here, the peptides on dextran were found to be closer (2.9 nm) to each other than in pHPMA (3.4) and hPG (3.7 nm). The larger distance in hPG might be related to the hypervalent morphology of this carrier, which possibly limits the proximity of attached ligands. Expected values of averaged (over time and atoms) radial distributions (correlating with normalized mean distances) of polymer atoms around peptide atoms clearly reveal a higher polymer-peptide proximity for the dextran system (1.23 nm) than for pHPMA (1.41 nm) and hPG (1.56 nm). Considering the statistical character of the underlying molecular ensemble, the time-averaged radial distribution function (rdf) values indicate a smaller ratio of the fraction of time steps with outstretched peptides (which are more accessible for binding with the tWW domain) and the fraction of time steps characterized by a contracted structure in case of peptides associated with the dextran polymer (Figure 4C). Thus, ligands attached to pHPMA or hPG are more often available for protein binding than those linked to dextran. As a consequence, multiple simultaneously outstretched peptides are even less likely to emerge in case of dextran in comparison with the other polymers. Moreover, after having bound the first protein and due to substantially smaller peptide end-to-end distances given with dextran, its next outstreched peptide will rather bind a free tWW domain of the same protein than of another one which clearly confirms the stoichiometric results. This binding mode is illustrated in Figure 5.

Another descriptor for the spatial arrangement that we denote as the peptide polymer's globularity was defined as the quotient of the volume under the multivalent ligand's solvent-accessible surface area (SASA) and the volume of the minimal sphere incorporating the entire molecule (Table 2). Not unexpectedly, the conformation of the peptide conjugate with the dendritic polymer hPG yields a significantly higher globularity (0.1) compared to those associated with pHPMA (0.04) or dextran (0.07). Regarding these two linear carriers only, the higher globularity of the dextran-based ligand is in good agreement with that material's peptide–polymer distance.

**Thermodynamic properties.** From a physical point of view, the significantly varying mean peptide–peptide and peptide–polymer distances are mainly attributed to molecular interactions between the involved atoms. For this reason we calculated non-bonded interaction energies between peptide atoms and both polymer and solvent atoms as the sum of van-der-Waals and electronic contributions (Table 2) While the interaction energies between peptides and solvents are, as expected, nearly identical for all three systems, the interaction of peptide atoms regarding polymer atoms amounts to substantially different values for the three carrier materials. With −913 kJ/mol dextran yielded the by far lowest energy compared with those peptides attached to the two high-stoichiometry polymers (−515 kJ/mol and −783 kJ/mol). Since lower energies correspond to preferential states, the interaction energy can be interpreted as a measure for a state's preference. In general, preferential states are characterized by (negative-signed) attractive forces dominating over (positive-signed) repulsive forces. Hence, according to these results, the peptide is more attracted by the dextran carrier than by the two others most likely causing the small expected polymer–peptide distance and possibly the small peptide end-to-end distances in case of dextran.

Finally, the molecular dynamics simulations of the peptide–polymer conjugates were compared with those of dimeric complexes with a bivalent binding mode in order to calculate the entropic loss of both the protein and of the peptide–polymer conjugates themselves (Table 3). Interestingly, in all three cases the major contribution to the entropic loss was on the side of the protein, the decrease in entropy on the polymer side was comparably small. Though bivalent binding modes are strongly favoured through enthalpic gain, the free energy gain is limited by the entropy loss, most likely caused by the flexibility of the linker and thus a larger number of alternative conformational states of the protein receptor.

**Table 3:** Calculated changes in entropy during binding of the multivalent polymeric ligands to the bivalent receptor by molecular dynamics simulations.

Binding partner	Entropy contribution *T*Δ*S* [kJ/mol]		
	
	pHPMA	hPG	dextran

Protein receptor	−14.91	−15.20	−14.74
Polymeric ligand	−0.67	−1.38	−0.92

**Σ**	−15.58	−16.58	−15.66

## Conclusion

All three investigated biocompatible polymers, namely linear poly(*N*-2-hydroxypropyl)methacrylamide (pHPMA), hyperbranched polyglycerol (hPG), and linear 2-carboxyethyldextran are suited for the construction of peptide–polymer conjugates, which can be used as potent multivalent ligands for a flexible protein–protein interaction site here exemplified by the tandem WW-domains of FBP-21. 2-Carboxyethyldextran furnished peptide–polymer conjugates with significantly higher binding affinity than the two other carriers. The observed binding modes of the three carriers were distinct. Dextran-based conjugates formed preferably bivalent, soluble complexes with a stoichiometry of <2 peptide ligands per protein binding site, while pHPMA and hPG formed colloidal suspensions/dispersions with stoichiometries >2 ligands per binding site. Molecular dynamics calculations suggested that conjugates with multivalently presented peptides on dextran occupy conformations in which two conjugated peptides are closer to each other and to the polymer backbone, corresponding to the calculated stronger peptide-polymer interaction. From the study it can be supposed that the simulated conformational space of the investigated peptide–polymer conjugates indeed correlates with the experimentally observed binding properties of the multivalent ligands. The construction and experimental investigation of further peptide–polymer conjugates will show, whether the results reported here will be helpful for the construction of even more potent multivalent and/or crosslinking ligands for protein–protein interaction sites and whether the ligands active in the protein binding assay can be further developed toward intracellularly delivered and intracellularly active PPI-inhibitors of the tandem WW-domain.

## Supporting Information

File 1 Experimental.
