# Supplementary material for: Peptide–polymer ligands for a tandem WW-domain, an adaptive multivalent protein–protein interaction: lessons on the thermodynamic fitness of flexible ligands
Source: Beilstein J Org Chem. 2015 May 18;11:837–47. doi: 10.3762/bjoc.11.93 (PMC4464424; doi:10.3762/bjoc.11.93)
Supplement: File 1 — Experimental. [file Beilstein_J_Org_Chem-11-837-s001.pdf]

Supporting Information

for

Peptide–polymer ligands for a tandem WW-domain, an adaptive multivalent protein–protein interaction: lessons on the thermodynamic fitness of flexible ligands

Katharina Koschek[1,2,3], Vedat Durmaz[4], Oxana Krylova[2], Marek Wieczorek[1,2], Shilpi Gupta[1], Martin Richter[1,2], Alexander Bujotzek[4], Christina Fischer[1], Rainer Haag[1], Christian Freund[1,2], Marcus Weber[4], and Jörg Rademann*[1,2]

Address: [1]Institute of Pharmacy & Institute of Chemistry and Biochemistry, Freie Universität Berlin, Königin-Luise-Str. 2+4, 14195 Berlin, Germany, [2]Department of Medicinal Chemistry, Leibniz Institut für Molekulare Pharmakologie, Robert-Rössle-Str. 10, 13125 Berlin, Germany, [3]current address: Fraunhofer Institute for Manufacturing Technology and Advanced Materials (IFAM), Wiener Str. 12, 28359 Bremen, Germany and [4]Konrad-Zuse-Zentrum für Informationstechnik Berlin, Numerical Analysis and Modelling, Takustr. 7, 14195 Berlin, Germany

Email: Jörg Rademann - joerg.rademann@fu-berlin.de

*Corresponding author

Experimental

Materials and methods

All moisture-sensitive reactions were performed in flame-dried Schlenk or round bottom flasks and were carried out under a nitrogen atmosphere. Dry solvents such as THF, DCM, DMF stored over molecular sieves were purchased from Fluka, and Aldrich as dry solvents and were used as received. Other solvents were of HPLC or reagent grade quality and obtained from Sigma-Aldrich. Acetonitrile for use in HPLC and HPLC-TOF analysis was purchased from Fisher Scientific as HPLC grade. Water for HPLC was purified applying a Milli-Q® system from Millipore. Deuterated solvents for NMR measurements were purchased either from Deutero or Euriso-Top. Spectra/Por 3 (3500 Da cut-off) membrane used for dialysis was obtained from Fisher Scientific and the benzoylated dialysis tubing (2000 Da cut-off) from Sigma Aldrich. Sephadex G25 (PD10) was obtained from GE Healthcare. All other used reagents were obtained from Sigma-Aldrich, Fluka and Merck and were used without purification unless otherwise noted.

Proton nuclear magnetic resonance (^{1}H NMR) and carbon nuclear magnetic resonance (^{13}C NMR) spectra were recorded on a Bruker AV 300 MHz instrument or on a JEOL 400 MHz instrument (Spectrometer ECX400) in deuterated solvents. High resolution mass spectrometry (HRMS) measurements were conducted with an Agilent 6220 ESI-TOF mass spectrometer in combination with a liquid chromatography workstation of Agilent 1200 series with Zorbax Eclipse XDB-C18 Rapid Resolution HT 50 (1.8 µm, 4.6 x 50mm) column from Agilent Technologies. The eluents water and acetonitrile (ACN) with 0.1 % formic acid were used in a linear gradient (5–99 % ACN in 2.5 min).

Liquid Chromatography–Ion Trap Mass spectrometry (LC-Q) measurements were performed with a liquid chromatography workstation of Thermo Finnigan equipped with a PDA-UV-Detector and a C18-ec column from Macherey-Nagel GmbH & Co KG in combination with a ion trap mass spectrometer. The eluents water and ACN with 0.1% formic acid were used in a linear gradient (5–99% in 8 min).
The peptide content of the peptide-polymer conjugates was determined by GC amino acid analysis (Genaxxon bioscience GmbH, Ulm, Germany) and high-sensitivity isothermal titration calorimetry (ITC) experiments were performed on the MicroCal iTC200 instrument (Malvern Instruments, UK).

Preparation of the polymeric ligands

Synthesis of peptide P1 and P2

P2: CGPPPRGPPPR-NH2

P1: GPPPRGPPPR-NH2

The peptides **P1** and **P2** were prepared by Fmoc-chemistry based solid phase synthesis as described earlier [1]. All reactions were performed in polypropylene syringes with adapted polyethylene frits and caps (Roland Vetter Laborbedarf OHG, Ammerbuch). In a 5 mL syringe Rinkamide-resin (500 mg, 0.310 mmol, 1.0 equiv) was swollen in DCM and the solvent was removed by filtration. To a solution of the appropriate Fmoc-protected amino acid (1.55 mmol, 5.0 equiv) in DMF 1-hydroxybenzotriazole (HOBt) (244 mg, 1.55 mmol, 5.0 equiv) and N,N´-diisopropylcarbodiimide (DIC) (240.4 µl, 0.806 mmol, 5.0 equiv) were added. The resulting solution was added to the resin and the mixture was shaken for 3 h before the solvent was removed and the resin was washed with DMF and DCM. Kaiser Test was applied to proof the completeness of the coupling. Before the next coupling step

the Fmoc group was removed by treating the resin with a piperidine solution (20% piperidine in DMF, 1 x 1 min, 2 x 10 min). Cleavage of the peptide from the resin was performed with a cleavage cocktail (TFA/H_2O/thioanisol/EDT/TIS 87:5:5:2:1). The cleaved peptide was precipitated in cold diethyl ether and purified by HPLC.

Purification:

P2: HRMS: m/z: calc. for $C_{49}H_{79}N_{18}O_{11}S$: 1129.5996, found: 1129.6174 $[M+H]^{+}$, (Δ 15.75 ppm).

P1: LC-Q: t_R = 0.28 min, m/z: 1026.85 $[M+H]^{+}$.

Synthesis of poly(HPMA-co-S-benzylthioestermethacrylamide) (NCL-polymer)

The NCL-polymer was prepared as described earlier by Koschek et al. [1] with the following specifications Mn = 13.3 kDa, PDI = 1.2; thioester loading: 0.87 mmol/g.

Synthesis of Maleimidoethyl amidoethyl dextran

Maleimidoethyl amidoethyl dextran was prepared as described earlier by M. Richter et al. [2]. For the preparation of peptide–polymer conjugates three different maleimidated-functionalized dextrans were used:

- 40 kDa MA-dextran exhibiting 25 maleimide groups
- 10 kDa MA-dextran exhibiting 13 maleimide groups
- 10 kDa MA-dextran exhibiting 5 maleimide groups

Synthesis of maleimidated hyperbranched polyglycerol (PG-Mal)

Hyperbranched polyglycerol (PG): Polyglycerol (PG) (Mn = 10.000 g/mol, Mw/Mn = 1.6) using 1,1,1- tris(hydroxylmethyl)propane (TMP) as initiator as described by A. Sunder et al. [3,4]

Maleimidated hyperbranched polyglycerol (PG-Mal): To an ice-cold solution of PG (50 mg, 0.68 mmol) in *N*,*N*-dimethylformamide (1 mL) were added triethylamine (71 µL, 0.51 mmol) and a solution of 3-maleimido propanoyl chloride [prepared in situ: to a solution of *N*-maleoyl-β-alanine (69 mg, 0.41 mmol) in $CHCl_3$ (1 mL) was added thionyl chloride (74 µL, 1.01 mmol) and the mixture was refluxed for 6 h; the solvent was then removed in vacuum and the resulting yellow oil was redissolved in $CHCl_3$ (1 mL) and cooled to 0 °C in $CHCl_3$. The reaction mixture was stirred under nitrogen for 24 h. The solvent was then removed in vacuo and the resulting yellow viscous oil was purified by dialyzing with 2000 Da MWCO dialysis tubing against MeOH to yield the maleimidated PG (50 mg, 84%, degree of functionalization: 6 % equals 6 maleimide groups / PG (approximation from NMR).
^{1}H NMR (400 MHz, methanol-d_3, 25 °C): δ = 2.68 (s, 2 H, $COCH_2$), 4.26-3.44 (m, 52 H, PG-protons), 6.86 (s, 2 H, olefinic protons of maleimide).

Peptide-polymer conjugates – Native Chemical Ligation (NCL)

The native chemical ligation of the NCL-polymer poly(HPMA-co-S-benzylthioestermethacrylamide) and an appropriate cysteinylated peptide was conducted as described earlier.[1]

pHPMA-**1:** Briefly, the NCL polymer (11 mg, 9 µmol, 1.0 equiv; thioester loading: 0.87 mmol/g) and the peptide **P2** (17.8 mg, 12.0 µmol, 1.3 equiv) thiophenol (1% v/v) yielded the product as a white solid (8.6 mg; yield: 58%, peptide loading 0.35 mmol/g corresponds to a loading of 6 peptides/polymer chain).

pHPMA-**2:** Briefly, the NCL-polymer (5.0 mg, 4.6 µmol, 1.0 equiv; thioester loading: 0.93 mmol/g), and peptide **P2** (8.0 mg, 5.2 µmol, 1.2 equiv) yielded the product as a white solid (4.7 mg; yield: 72%, peptide loading 0.42 mmol/g corresponds to a loading of 9 peptides/polymer chain).

Peptide–polymer conjugates – Thiol-Michael reaction (Thiol-maleimide Click reaction)

hPG-**1:** A solution of maleimidated hyperbranched polyglycerol (PG-Mal6) (6 mg, 8 µmol, 1.0 equiv, maleimide loading: 1.15 mmol/g) in 50 mM sodium phosphate buffer (10 mg/mL, pH 7.0) containing 5 mM EDTA was degassed for 30 min with nitrogen before the addition of the peptide **P2** (15.4 mg, 10.0 µmol, 1.3 equiv; 2 mg/mL) dissolved in phosphate buffer containing 50 mM TCEP. The reaction mixture was stirred vigorously overnight at room temperature. Mercaptoethanole (0.73 µl, 10 µmol, 1.3 equiv) was added and the mixture was stirred for additional 8 h. The reaction mixture was thereafter purified by gelfiltration applying Sephadex® G-25 (PD10 column) and dialysis (3500 Da cut-off, 48 h) and then lyophilized. The product was obtained as a pale-coloured powder (8.1 mg yield: 99%; peptide loading: 0.260 mmol/g corresponds to a loading of 3 peptides / polymer chain).

hPG-**2:** was prepared accordingly to hPG-1. Briefly, the reaction mixture of PG-Mal6 (5.2 mg, 5.8 µmol, 1.0 equiv, : 1.15 mmol/g) and CP1 (11 mg, 7.5 µmol, 1.2 equiv) yielded the product as a pale-coloured powder (7.8 mg, yield: 99%; peptide loading: 0.346 mmol/g corresponds to a loading of 3-4 peptides / polymer chain).

Dex-**1:** Maleimidated dextran (10 mg, 0.861 µmol, 1.0 equiv, 10 kDa dextran; 5 maleimido residues) and peptide **P2** (6.3 mg, 5.6 µmol, 6.5 equiv) were dissolved in a mixture of phosphate buffer (50 mM, pH 7.0) and formamide (buffer/formamide 3:1) and stirred overnight. The reaction mixture was purified by gelfitration applying Sephadex® G-25 (PD10 column) and dialysis (3500 Da cut-off, 48 h) and then lyophilized. The product was obtained as colourless powder (13.3 mg, yield: 90 %; peptide loading 0.223 mmol/g corresponds to a loading of 3 peptides / polymer chain).

Dex-**2** was prepared according to Dex-1: Briefly, maleimidated dextran (10 mg, 0.771 µmol, 1.0 equiv, 10kDa, 13 maleimido residues) and peptide **P2** (4.0 mg, 3.5 µmol, 5 equiv) yielded the product as colorless powder (11 mg, yield: 73 %; peptide loading: 0.348 mmol/g corresponds to a loading of 6 peptides / polymer chain).

Dex-**3:** was prepared accordingly to Dex-1. Briefly, maleimidated dextran (10 mg, 0.861 µmol, 1.0 equiv, 40kDa dextran) and peptide **P2** (10 mg, 8.86 µmol, 5 equiv) yielded the product as white solid (10 mg, yield: 80%; peptide loading: 0.183 mmol/g corresponds to a loading of 8 peptides / polymer chain).

Isothermal Titration Calorimetry (ITC) Experiments

Sample preparation

All samples for ITC were prepared with the phosphate buffer used for protein purification. A 10 mM stock solution of the peptide polymer conjugate in PBS was prepared and adjusted to pH 7.4 with a 50 mM NaOH solution in PBS. Aliquots of the stock solution were diluted to the final concentration of 1.5-2 mM with PBS.

Protein expression

Proteins were expressed as His- or GST-tagged fusion constructs in E.coli BL21 (DE3), purified by the corresponding affinity matrices, thrombin cleaved and applied to a gel filtration column (superdex-75) at 50 mM phosphate, 150 mM NaCl, 1 mM EDTA, pH 6.5 or 1xPBS, 1 mM EDTA, pH 7.4. Proteins were concentrated by Centrifugal Filter Units (regenerated cellulose, 3 kDa cut-off, Millipore) if necessary.

ITC measurements

2 µl aliquots of peptide-polymer conjugates were titrated into a 50-70 µM protein solution inside the calorimeter cell (200 µl) while the reaction mixture was continuously stirred at 1000 rpm. Ligand concentration in the titration syringe was typically in 20-time excess corresponding to protein. Time intervals between injections were adjusted to 240 s which was sufficient for the heat signal to return to baseline level. In control experiments peptide-polymer conjugates were injected into buffer which resulted in small and constant heat of dilution. The heats of dilution were subtracted from the heats determined in the corresponding protein-ligand binding experiments. Automated baseline adjustment and peak integration were accomplished with the public-domain software NITPIC [5]. Estimation of best-fit parameter values by nonlinear least-squares fitting and calculation of 95% confidence intervals were performed with the public-domain software SEDPHAT [6]. Polymer-attached peptides were considered as monovalent ligands, and the tandem WW protein as receptor molecule with two similar independent binding sites. Binding was analysed in terms of simple hetero-association model. Corresponded calculation gives the microscopic binding constants that characterise the binding affinity of peptide moieties on polymer matrix, as well as enthalpic and entropic contribution to the binding process. Stoichiometry parameter (N) gives the number of peptide molecules bound in the complex with one tandem WW protein. The entropy error was calculated according to the covariance propagation law:

$$\partial S^2 = \frac{1}{T^2} \times \partial H^2 + R^2 \times \left(\frac{\partial K}{K}\right)^2$$

Molecular modeling

After having had drawn, 3D-cleaned and exported the coordinates of all monomeric units using version 5.5.0.1 of the chemical drawing software MarvinSketch [7], the three polymers as well as the peptide sequence were built and connected with the aid of the molecular analysis module [8] of the visualization software Amira [9]. Force field parameters including partial atomic charges based on the am1bcc algorithm [10] were assigned by the Antechamber [11] program from the ambertools package version 12. In contrast to dextran and pHPMA, pPG building as well as parameterization was achieved using a recently described probabilistic pPG polymer building tool [12].

The multivalent polymer ligands were explicitly solvated according to the tip4pew water model [13]. In order to remove molecular strain and unphysical atomic overlaps, they first underwent an energy minimization routine using the conjugate gradient algorithm followed by two equilibration phases. Both consisted of 200.000 MD steps, thereby, representing different thermodynamic boundary conditions: constant volume during equilibration phase one and constant pressure during the second phase applying Berendsen's algorithm [14]. Positions of polymer and peptide atoms were restrained during both equilibration stages. The final state of each polymer ligand was taken as initial structure for three independent unrestricted 100ns production runs with, in analogy to the equilibration, a time step size of 2fs and a temperature coupled to 298K according to the velocity rescaling approach [15].

Liquid chromatography and mass spectrometry of P1 and P2

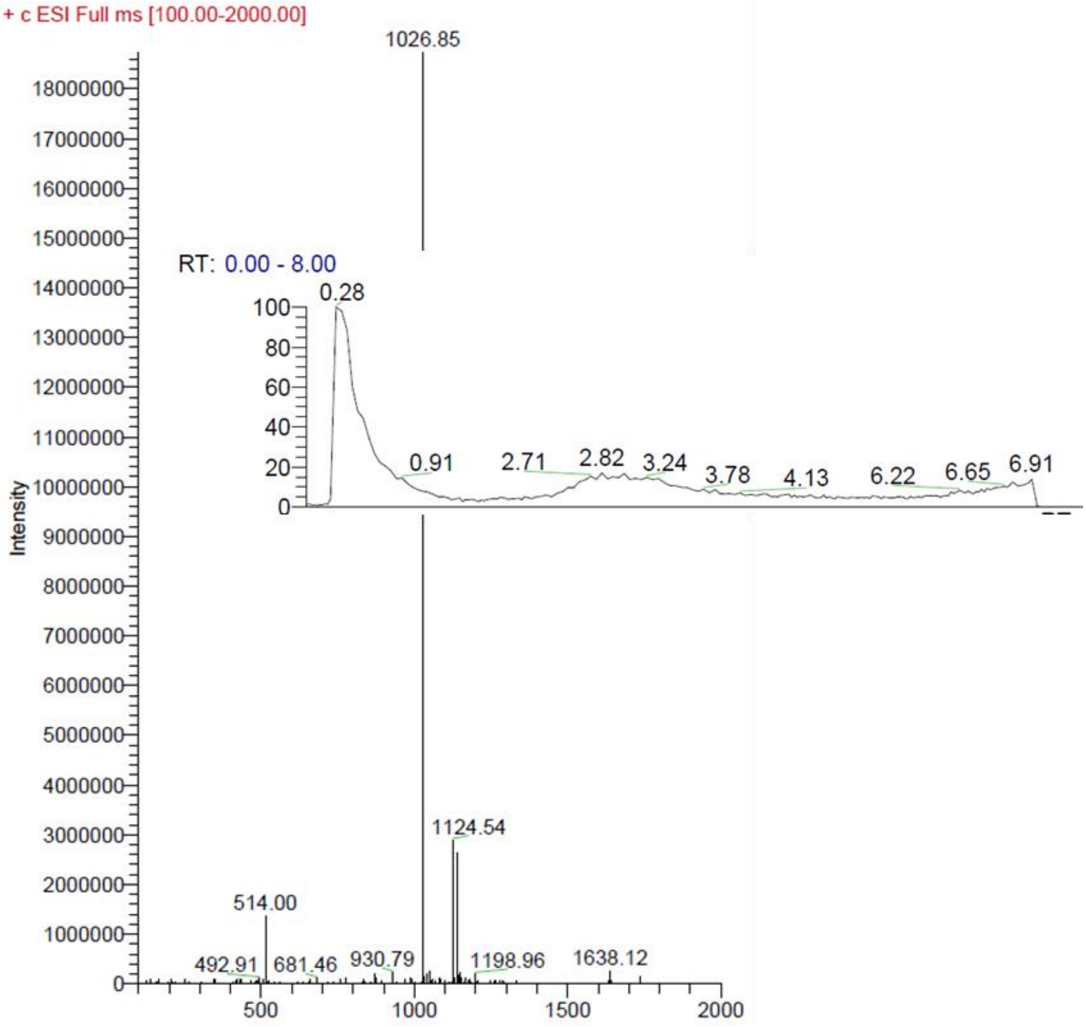

Figure S 1: LC-Q of peptide **P1**.

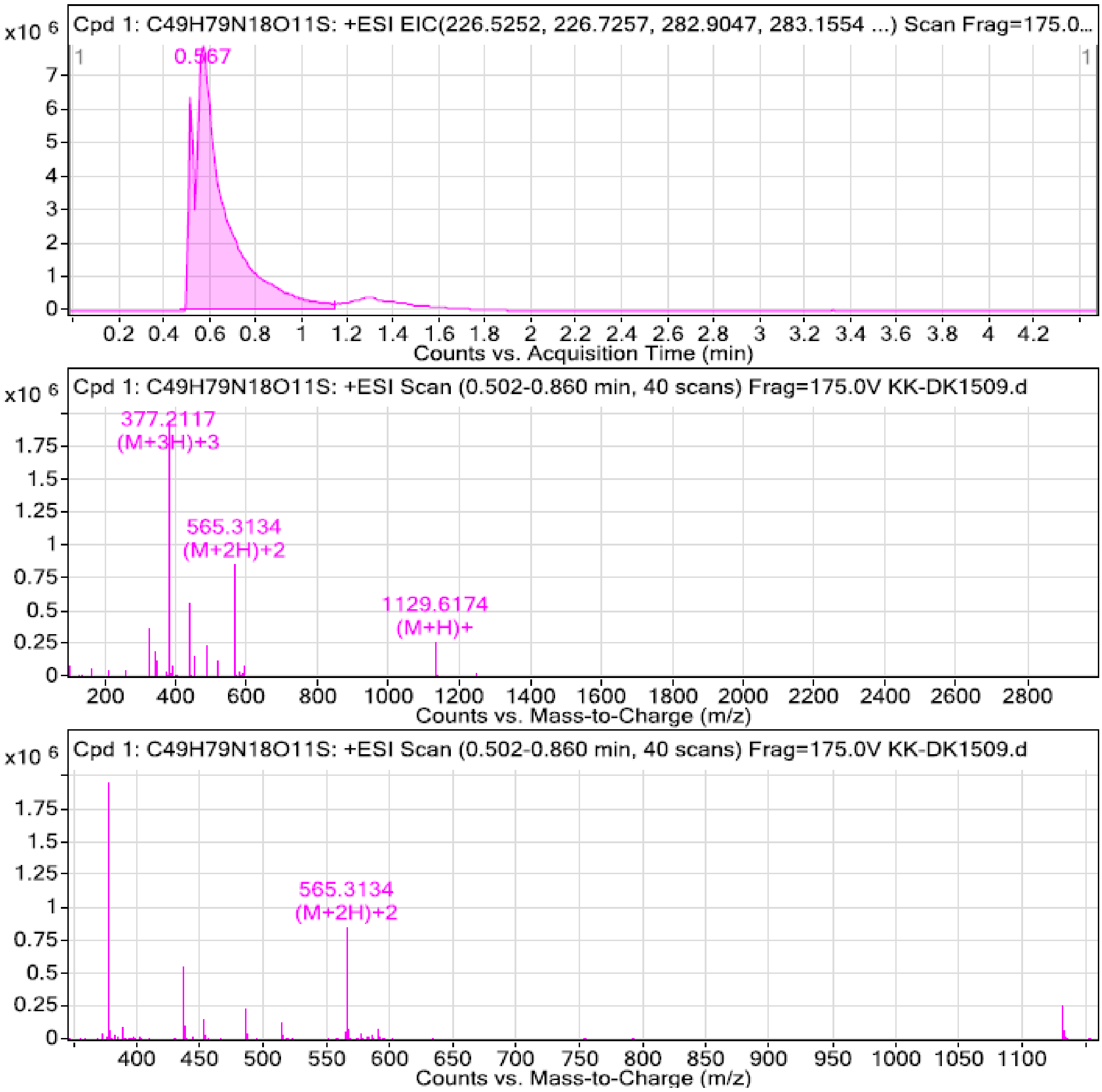

Figure S 2: HRMS of peptide **P2**.

^{1}H NMR of maleimidated PG

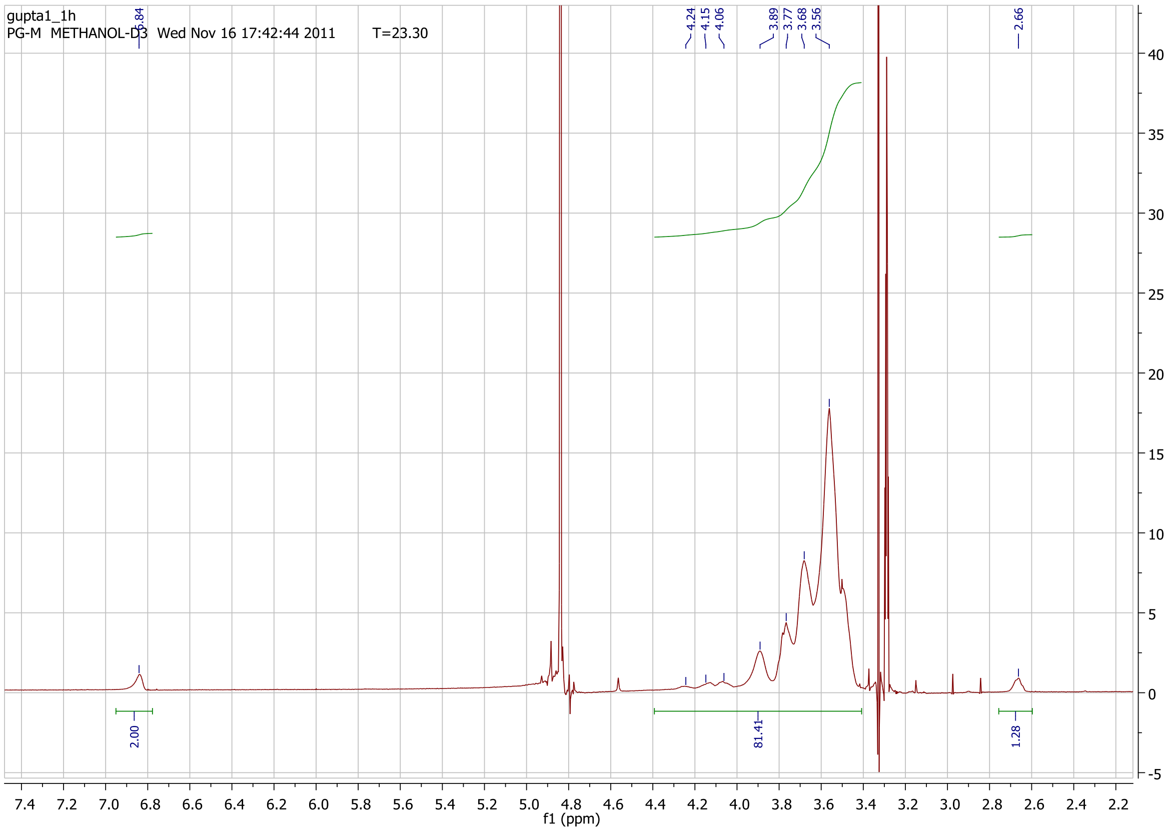

ITC curves for the interaction of peptide-polymer conjugates with FBP21

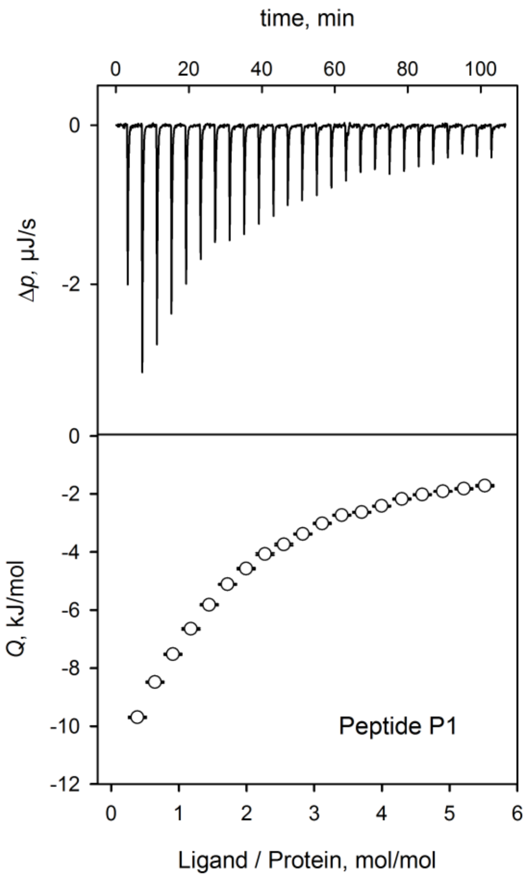
time, min
0 20 40 60 80 100
Δp, µJ/s
0
-2
Q, kJ/mol
0
-2
-4
-6
-8
-10
-12
Peptide P1
0 1 2 3 4 5 6
Ligand / Protein, mol/mol

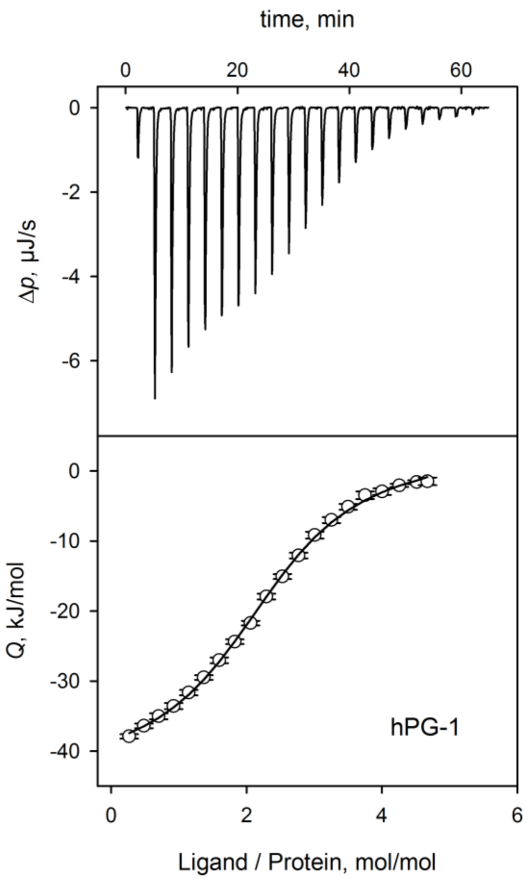
time, min
0 20 40 60
Δp, µJ/s
0
-2
-4
-6
Q, kJ/mol
0
-10
-20
-30
-40
hPG-1
0 2 4 6
Ligand / Protein, mol/mol

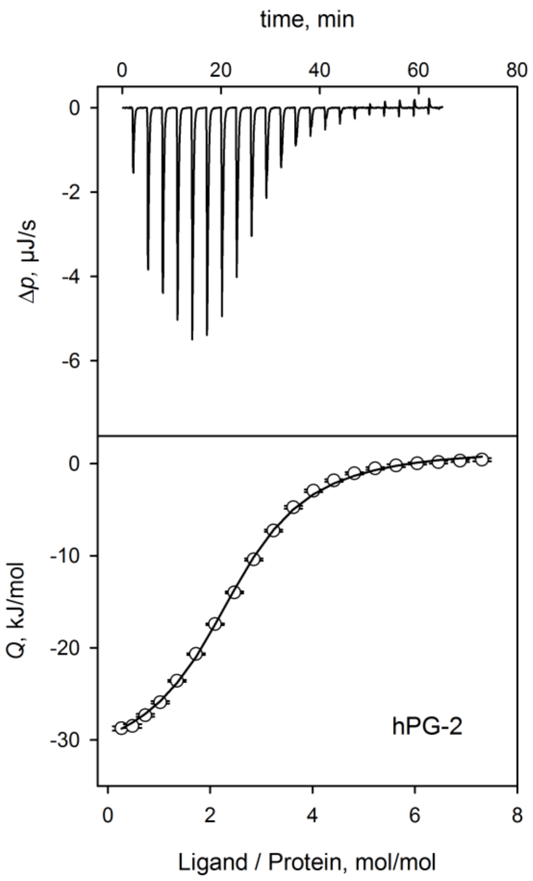
time, min
0 20 40 60 80
Δp, µJ/s
0
-2
-4
-6
Q, kJ/mol
0
-10
-20
-30
hPG-2
0 2 4 6 8
Ligand / Protein, mol/mol

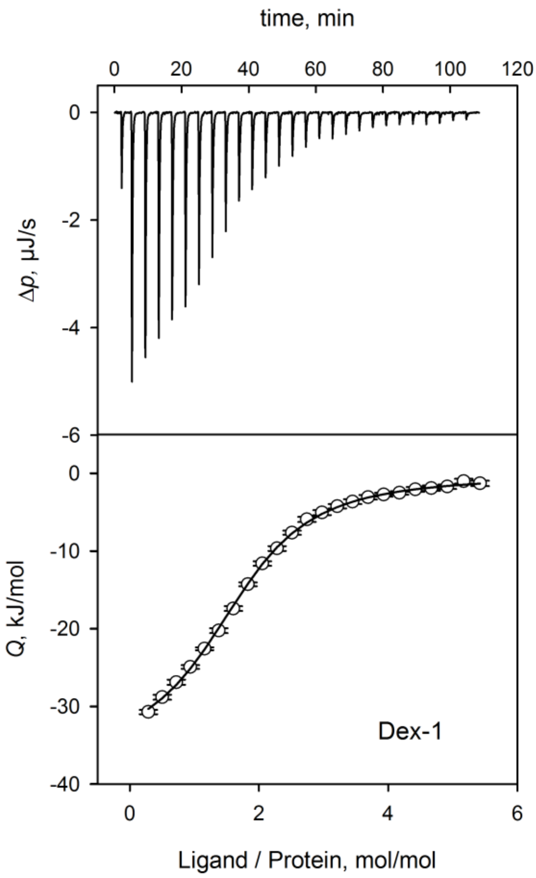
time, min
Δp, μJ/s
Q, kJ/mol
Dex-1
Ligand / Protein, mol/mol

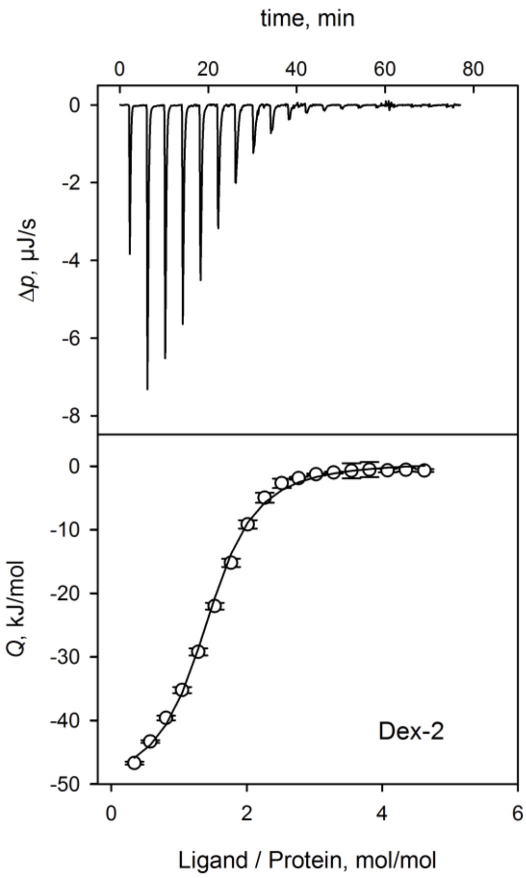
time, min
Δp, μJ/s
Q, kJ/mol
Dex-2
Ligand / Protein, mol/mol

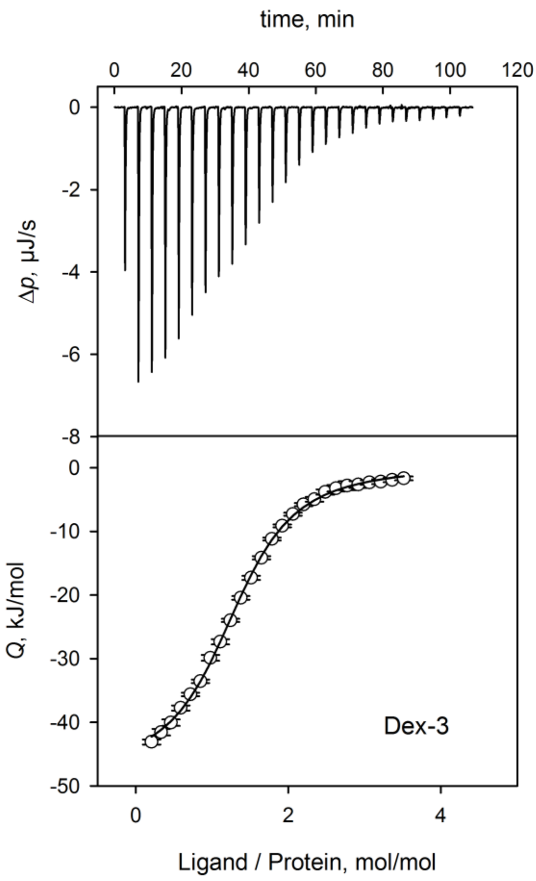
time, min
Δp, μJ/s
Q, kJ/mol
Dex-3
Ligand / Protein, mol/mol

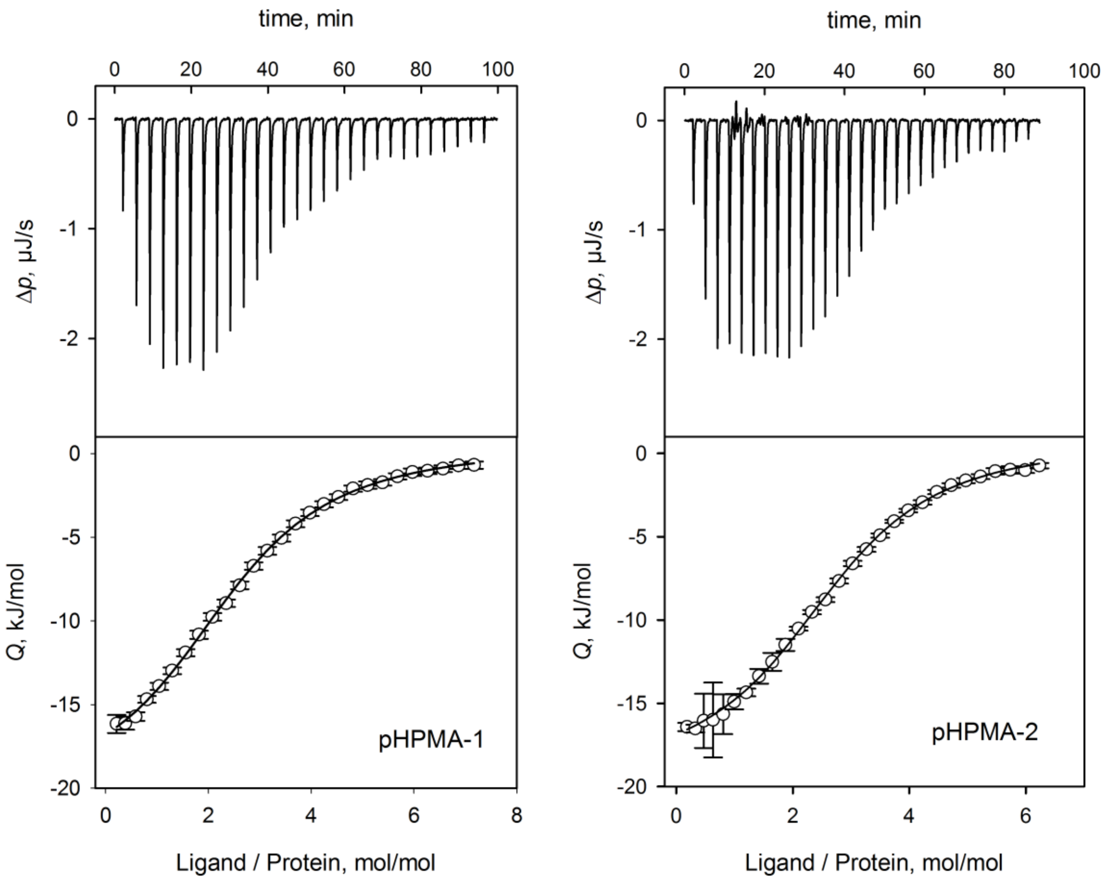
time, min
Δp, µJ/s
Q, kJ/mol
pHPMA-1
pHPMA-2
Ligand / Protein, mol/mol

References

(1) Koschek, K.; Dathe, M.; Rademann, J. *Chembiochem* **2013**, *14*, 1982–1990.

(2) Richter, M.; Chakrabarti, A.; Ruttekolk, I. R.; Wiesner, B.; Beyermann, M.; Brock, R.; Rademann, J. *Chem. Eur. J.* **2012**, *18*, 16708–16715.

(3) Sunder, A.; Hanselmann, R.; Frey, H.; Mühlhaupt, R. *Macromolecules* **1999**, *32*, 4240–4246.

(4) Sunder, A.; Mülhaupt, R.; Haag, R.; Frey, H. *Adv. Mater.* **2000**, *12*, 235–239.

(5) Keller, S.; Vargas, C.; Zhao, H.; Piszczek, G.; Brautigam, C. A.; Schuck, P. *Anal. Chem.* **2012**, *84*, 5066–5073.

(6) Houtman, J. O. N. C. D.; Brown, P. H.; Bowden, B.; Yamaguchi, H.; Appella, E.; Samelson, L. E. *Protein Sci.* **2007**, *16*, 30–42.

(7) http://www.chemaxon.com/products/marvin/marvinsketch/.

(8) Schmidt-Ehrenberg, J.; Baum, D.; Hege, H.-C. *IEEE Visualization 2002*; IEEE Computer Society Press, 2002; pp. 235–242.

(9) Stalling, D.; Westerhoff, M.; Hege, H.-C. Academic Press, 2004.

(10) Wang, J.; Wang, W.; Kollman, P. A.; Case, D. A. *J. Mol. Graph. Model.* **2006**, *25*, 247–260.

(11) Durmaz, V. *J. Comput. Aided. Mol. Des.* **2015**, *29*, 225–232.

(12) A. Jakalian, Jack, D. B.; Bayly,C. I. *J. Comput. Chem.* **2002,** *23,* 1623–1641.

(13) Horn, H. W.; Swope, W. C.; Pitera, J. W.; Madura, J. D.; Dick, T. J.; Hura, G. L.; Head-Gordon, T. *J. Chem. Phys.* **2004**, *120*, 9655–9678.

(14) Berendsen, H. J. C.; Postma, J. P. M.; Gunsteren, W. F.; DiNola, A.; Haak, J. R. *J. Chem. Phys.* **1984**, *81*, 3684–3690.

(15) Bussi, G.; Donadio, D.; Parrinello, M. *J. CHem. Phys.* **2007**, *126*, 1–8.
